# Structure and Intercalation of Cysteine–Asparagine–Serine Peptide into Montmorillonite as an Anti-Inflammatory Agent Preparation—A DFT Study

**DOI:** 10.3390/molecules29174250

**Published:** 2024-09-07

**Authors:** Carolina Barrientos-Salcedo, Catalina Soriano-Correa, Alfonso Hernández-Laguna, Claro Ignacio Sainz-Díaz

**Affiliations:** 1 Laboratorio de Química Médica y Quimiogenómica, Universidad Veracruzana, Veracruz C.P. 91700, Mexico; cabarrientos@uv.mx; 2 Instituto Andaluz de Ciencias de la Tierra, Consejo Superior de Investigaciones Científicas, Av. de las Palmeras, 4, 18100 Armilla, Granada, Spain; a.h.laguna@csic.es; 3 Unidad de Química Computacional, Facultad de Estudios Superiores Zaragoza, Universidad Nacional Autónoma de México, Iztapalapa, Mexico City C.P. 09230, Mexico

**Keywords:** tripeptide, anti-inflammatory drug, quantum mechanical calculations, controlled drug release, clay minerals, dipeptide crystal structure, IR spectroscopy

## Abstract

Peptides are receiving significant attention in pharmaceutical sciences due to their applications as anti-inflammatory drugs; however, many aspects of their interactions and mechanisms at the molecular level are not well-known. This work explores the molecular structure of two peptides—(i) cysteine (Cys)–asparagine (Asn)–serine (Ser) (CNS) as a molecule in the gas phase and solvated in water in zwitterion form, and (ii) the crystal structure of the dipeptide serine–asparagine (SN), a reliable peptide indication whose experimental cell parameters are well known. A search was performed by means of atomistic calculations based on density functional theory (DFT). These calculations matched the experimental crystal structure of SN, validating the CNS results and useful for assignments of our experimental spectroscopic IR bands. Our calculations also explore the intercalation of CNS into the interlayer space of montmorillonite (MNT). Our quantum mechanical calculations show that the conformations of these peptides change significantly during intercalation into the confined interlayer space of MNT. This intercalation is energetically favorable, indicating that this process can be a useful preparation for therapeutic anti-inflammatory applications and showing high stability and controlled release processes.

## 1. Introduction

It is well known that inflammation is a biological response of the immune system to events that are initiated by a complex sequence of biochemical processes produced by some chemical intermediaries. These biochemical processes can be triggered by a variety of factors, including traumatic injuries tissues, pathogens, cardiovascular, bowel disease, diabetes, rheumatoid arthritis, cancer damaged cells, and toxic compounds. Infectious agents, such as viruses and bacteria, are some of the most common causes of inflammation. Viruses give rise to inflammation by entering and destroying cells in the body; bacteria release substances called endotoxins that can initiate inflammation [1,2]. These factors may induce acute and chronic inflammatory responses in the heart, pancreas, liver, kidney, lung, brain, intestinal tract, and reproductive system, potentially leading to tissue damage or disease. Likewise, uncontrolled acute inflammation may become chronic, contributing to a variety of multifactorial diseases, such as cancer, cardiomyopathies, and neurodegenerative diseases. These pathologies and factors are only partially understood [2,3].

In this sense, an inappropriate immune response may give rise to a prolonged and damaging inflammatory response. Examples include allergic or hypersensitivity reactions in which either an environmental agent, such as pollen and pollution, or a food type that normally does not pose any threat to individuals, stimulates inflammation and autoimmune reactions, and where chronic inflammation is triggered by the body’s immune response. Equally, physical trauma, burns, and radiation injuries can damage tissues and bring about inflammation. The same inflammation processes can be caused by corrosive chemicals, such as acids, alkalis, and oxidizing agents. Several molecules, such as free radicals, carbohydrates, and polypeptides derived from the invading organisms at the site of the damaged tissues, can also produce inflammatory effects, while others are the products of cells participating in the inflammatory response [4]. Small peptides are receiving significant interest as compounds for anti-inflammatory therapeutics [4].

The study of these peptides at the molecular level as drugs is not well established. Some studies have shown that certain electron delocalization along the backbone of peptides and proteins can be caused by a change in the three-dimensional chemical structure or substituent group in the side chain of the amino acids, thereby changing their properties [5,6]. In this sense, substitutions or changes are significantly important for modifying the peptide conformation and even the protein folding process, which is controlled by several factors, such as charge, hydrophobicity, pKa, and steric effects. These substitutions or changes could, however, affect the chemical reactivity of peptides and proteins and lead to a therapeutic effect [7,8,9]. Furthermore, the conformations adopted by a peptide or protein also depend on its intermolecular interactions, rotation angles, charge, polar interaction networks, and its ability to form hydrogen bonds with other side chain residues or backbone atoms that might contribute to stabilizing the structure. Such conformations play an important role in the physicochemical properties and chemical reactivity of peptides and proteins [5,7].

Amino acids are the building blocks of peptides and proteins. The interactions of amino acids with water, metal ions, protons, anions, and solid surfaces have been extensively investigated, providing useful information about their physicochemical properties, conformational distributions, and binding affinities. In this sense, it is important consider that the amino acids in aqueous solutions predominate in the zwitterion form. Zwitterions show different physicochemical properties and, consequently, their biological and pharmaceutical functions may change [10,11,12,13]. Clay minerals are the surface of choice when seeking to intercalate drugs or to protect amino acids from degradation [14,15,16,17].

The clay mineral montmorillonite (MNT) has been extensively studied and used in various industrial and pharmaceutical applications due to its swelling and high adsorption properties, which include the polymerization of organic molecules, nucleic acids, amino acids, and therapeutic applications [18,19,20,21,22,23]. Thus, the high adsorption capacity of MNT contributes to increasing drug entrapment and facilitates the prolonged release of drugs [19,20]. This clay mineral could be employed to formulate diverse drug delivery systems to control or improve the pharmaceutical disadvantages of certain drugs, including low solubility, dissolution rates, absorption, and poor pharmacokinetic properties (low bioavailability and short biological half-life) [21,22,23,24]. Likewise, some studies on hybrid polymer composites have shown that the presence of intercalated amino acids in MNT improves the mechanical properties of materials [25,26]. Previous studies have found that the tripeptide Cys-Asn-Ser (CNS) shows promising anti-inflammatory properties [27]. However, these oligopeptides have limited stability because they are not large enough to form secondary and tertiary structures [8]. The encapsulation of these peptides into the confined interlayer space of MNT can stabilize them and increase their therapeutic applications [28,29].

Driving forces for the behavior of these materials are based on the interatomic and intermolecular interactions at the atomic scale. These interactions can be studied by using theoretical calculations at the molecular and atomic scales. Previous studies on drug–mineral systems using this approach have proven it to be a useful tool for understanding some experimental behavior [29,30]. The main aims of this work are, by means of DFT calculations, to determine the molecular structure of these small peptides in order to analyze the intermolecular interactions in the crystal structure of one dipeptide, the experimental and theoretical IR spectroscopic properties, and the main interactions between peptides and MNT during the intercalation process.

## 2. Results and Discussion

### 2.1. Molecular Structure of Peptides

**Figure 1:**
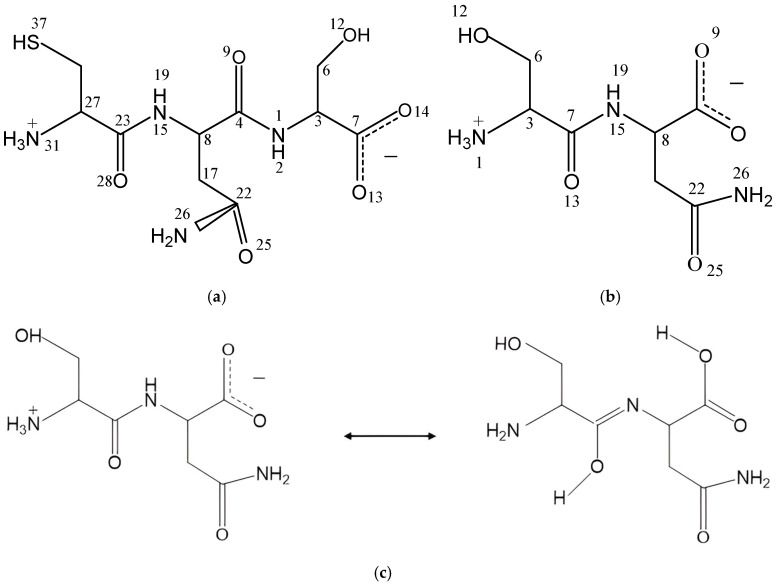
The tripeptide Cys-Asn-Ser (CNS) (**a**) and dipeptide Ser-Asn (SN) (**b**) structures in zwitterion forms, and tautomerization reaction of SN (**c**).

The zwitterion forms of the CNS and SN peptides were considered in our models since these forms exist in aqueous dissolution (Figure 1). We selected the CNS conformer with the lowest energy reported in a previous conformational analysis of CNS [8,12]. These models were optimized at the DFT level with Gauss and CASTEP codes (see later). In CNS, the terminal ammonium group is in the cysteine unit and the carboxylate group is in the serine unit, whereas in SN, the ammonium group is in the serine unit and the carboxylate is in the Asn unit. The optimized CNS isolated molecule maintained the zwitterion form by using both codes. The bond lengths of the polar bonds (N-H, S-H, C=O, C-NH_3_^+^, C-NH_2_) calculated with CASTEP are slightly larger than those calculated using Gauss. Some changes in the dihedral angles were observed between the optimized structures with both codes (Tables S1–S3).

**Figure 2:**
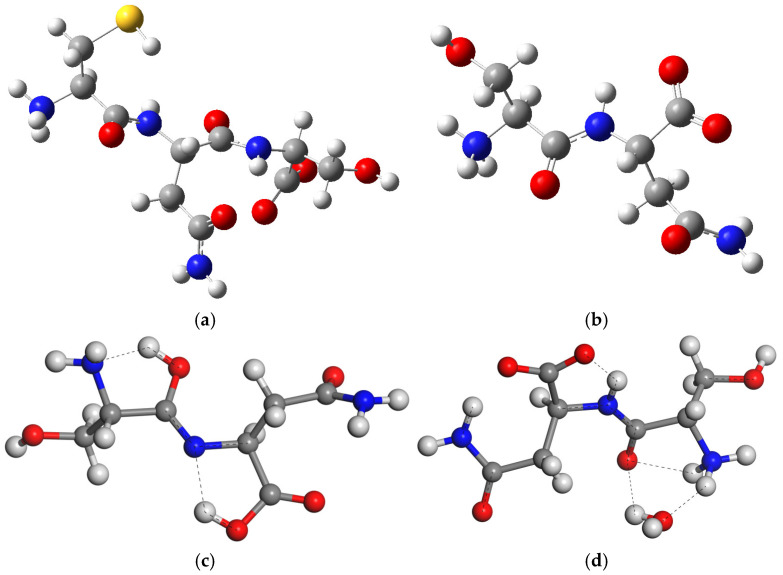
Optimized structures of isolated molecules of CNS tripeptide (**a**) and SN dipeptide (**b**) in zwitterion form, the most stable tautomer of SN (**c**), and the SN zwitterion with one water molecule SNw (**d**). The S, C, N, O, and H atoms are in yellow, grey, blue, red, and white colors, respectively, maintaining this criteria in this work. The dashed lines highlight the main hydrogen bonds.

In dry conditions, the tautomer form of the isolated SN molecule is 21.2 (Gaussian) and 87.07 (CASTEP) kcal/mol more stable than the zwitterion form, following the reaction in Figure 1c. This tautomer has a HO-C=N-C moiety formed by a migration of one H atom coming from the ammonium group of the zwitterion to the vicinal carbonyl group, and the simultaneous migration of one H atom of the NH group to the vicinal carboxylate group (Figure 1c). Two intramolecular hydrogen bonds exist in this tautomer (Figure 2), one between the imino group and the vicinal amino group d(OH…NH_2_) = 1.834 Å, and other between the carboxylic group and the imino group d(COH…NC) = 1.808 Å. One C-N bond length changes from 1.475 to 1.282 Å, forming the imino bond, and the carbonyl bond changes from 1.220 to 1.341 Å, forming the hydroxyl group (Table S4).

However, the zwitterion is 15.7 (Gaussian) kcal/mol more stable than the tautomer in aqueous media. We created a model of the SN zwitterion with one water molecule attached like in the SN crystal. The presence of this water molecule (CASTEP) avoids the formation of this tautomer due to the intermolecular hydrogen bonds between the water molecule and SN, d(H_2_O…H_3_N) = 1.784 Å and d(HOH…OC) = 2.309 Å. Nevertheless, there is a partial tendency to form a structure similar to a tautomer with an intramolecular hydrogen bond between the NH and the carboxylate O atom, d(NH…OC) = 1.608 Å, with a shortage of the CN bond from 1.463 to 1.321 Å and an elongation of the CO bond from 1.230 to 1.251 Å (Figure 2d). 

**Figure 3:**
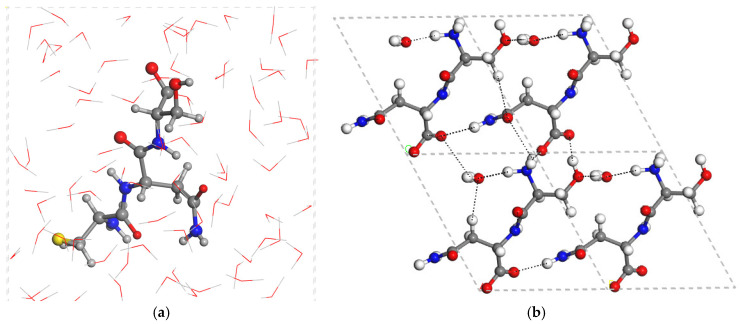
Optimized (CASTEP) zwitterion structure of CNS hydrated with 80 water molecules (**a**), and the crystal structure of the SN dimer with the crystallization water molecule (**b**). The dashed lines highlight the main hydrogen bonds.

The hydrated boxes with 80 water molecules of CNS (CNS_80w) in zwitterion form show hydrogen bonds between the water molecules and the main functional groups of CNS (Figure 3). The hydration box has 80 water molecules per unit cell (80w). Then, we generated an additional periodical box with only 80 water molecules per unit cell, optimizing it under the same computational conditions as CNS_80w. Considering the isolated molecule of CNS, we calculated (CASTEP) the hydration energy, *E*_hyd_, of CNS as:

*E*_hyd_ = *E*_CNS_80w_ − *E*_CNS_ − *E*_80w_ (1)

The hydration energy of CNS turned out to be −77.41 kcal/mol per peptide molecule, indicating that the solubilization of CNS in water is energetically favorable.

### 2.2. SN Crystal Structure

The crystal structure of SN has one crystallization water molecule, and SN is in zwitterionic form [31]. This structure was fully optimized with CASTEP by relaxing the atom positions and cell parameters, yielding *a* = 4.74 Å, *b* = 7.50 Å, *c* = 8.75 Å, α = 116.0°, β = 90.4°, and γ = 105.2°, which are quite close to the experimental values (*a* = 4.7 Å, *b* = 7.51 Å, *c* = 8.56 Å, α = 115.7°, β = 90.3°, and γ = 105.0° [31]). Despite the lack of experimental powder X-ray diffraction data, we simulated this diffractogram from the experimental data of the monocrystal and compared it with our calculated structure (Figure S1). Most of the reflection peaks of the optimized structure are consistent with the experimental ones. However, some minor differences can be observed, as follows: At the (002) reflection 22.9 (theoretical)–23.2 (experimental) 2θº; at the (0,2,−2) reflection 26.6 (theoretical)–26.9 (experimental); at the (1,1,−2) reflection 29.1 (theoretical)–29.4 (experimental); and at the (0,1,−3) reflection 30.7 [*d*(hkl) = 2.91 Å] (theoretical)– 31.4 [*d*(hkl) = 2.85 Å] (experimental). These differences could come from the experimental crystal being slightly more compressed (smaller *d*(hkl) values) than the calculated crystal. This can be observed from the smaller values of the *a* and *c* cell experimental parameters than those of the calculated parameters. Nevertheless, these differences represent only 1.3–2% and do not come from a non-real experimental pattern but a simulated experimental diffractogram that can change depending on the experimental conditions.

The main intermolecular interactions in the SN crystal lattice are one hydrogen bond between the NH_2_ group and the carboxylate, d(NH…O) = 1.964 Å. Additionally, this carboxylate forms two additional strong hydrogen bonds: one with the ammonium group, d(NH…O) = 1.766 Å; and another one with the COH group, d(OH…O) = 1.621 Å. The amide carbonyl group forms two additional hydrogen bonds: one with the NH_2_ group, d(NH…O) = 1.903 Å; and another one with the NH group, d(NH…O) = 1.949 Å. The crystallization water molecule acts as a intermolecular bridge forming hydrogen bonds with the ammonium group, d(NH…OH_2_) = 1.703 Å, and the serine COH groups, d(HOH…O(H)-C) = 1.831 Å. The conformation of the isolated molecule changes when it is crystallized as a monohydrate. The central amide group is coplanar with a dihedral angle H-N-C=O of 4.5°, and one carboxylate O atom is coplanar with the vicinal NH group with a dihedral angle O-C-C-N of 9.0°, whereas the carbonyl bond is twisted with respect to the ammonium group. O=C-C-NH_3_ = 58.1°, and the hydroxy O-H bond is twisted with respect to the vicinal N atom, HO-C-C-N = 56.2°.

In this crystal structure, the presence of the crystallization water molecule is critical, being responsible for the packing between SN molecules. Therefore, a single-molecule model of a SN zwitterion joined to one water molecule (SNw) in an isolated box was created and optimized in the same conditions. The resulting model maintained the hydrogen bond interaction between SN and the water molecule. Additionally, we can calculate the packing energy *E*_pack_ of the SN crystal (SNwcryst) as the difference in the single molecule SNw:

*E*_pack_ = *E*_SNwcryst_ − *E*_SNw_ (2)

Hence, the calculated packing energy of SNwcryst is −89.12 kcal/mol per unit cell, indicating that the changes introduced by the SN and the water molecule in the crystal produce intermolecular interactions between the SN zwitterions in addition to the interactions with the water molecule. This result corroborates that the crystallization of SN is energetically favorable.

### 2.3. CNS Intercalated in Montmorillonite

The mineral MNT is usually hydrated in nature, and the amount of water is variable depending on the experimental conditions of environmental moisture in a space [16]. In this work, the crystal structure was a 3 × 2 × 1 supercell of an MNT model, with 12 water molecules coordinating the Na cations in the interlayer space. This amount of water is a compromise between the minimal size model and the solvation of each interlayer cation by two water molecules, which yielded good results in previous work [17]. This structure was fully optimized by relaxing the atomic positions and lattice cell parameters with the CASTEP code, yielding a crystal structure with the following cell parameters: *a* = 15.70 Å, *b* = 18.21 Å, *c* = 14.55 Å, α = 91.9°, β = 106.5°, and γ = 89.8°.

**Figure 4:**
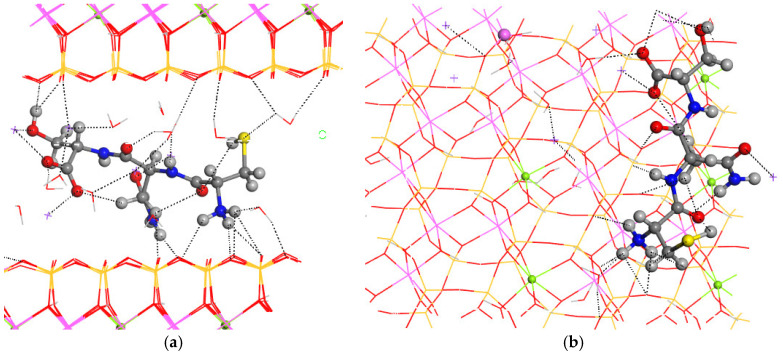
Intercalation of the CNS tripeptide into the MNT interlayer space. Views from 010 (**a**) and 001 (**b**) crystallographic planes. The main intermolecular interactions are described with dotted lines. The Si, Al, Mg, and Na atoms are in ochre, pink, green, and fuchsia, respectively. The S, C, N, O, and H atoms are in yellow, grey, blue, red, and white colors, respectively.

For the final step of the intercalation process, the CNS tripeptide was placed in the center of the interlayer space of MNT. The optimization of this hybrid material yielded a crystal structure similar to the pristine one, increasing only the *c* axis, and subsequently, the interlayer space (*a* = 15.70 Å, *b* = 18.22 Å, *c* = 17.23 Å, α = 88.5°, β = 108.6°, and γ = 89.7°) (Figure 4). The conformation of CNS changed completely after the intercalation in MNT, where the carbonyl and NH groups are coplanar and the ammonium and amine groups are twisted toward the basal O atoms of the mineral surface (Figure 4). The H atom of the hydroxyl group is not oriented to the carboxylate group as in the CNS molecule, but this intramolecular hydrogen bond is destroyed and the OH group is oriented to the hole formed by the basal tetrahedral O atoms of the opposite side of the mineral surface. The CNS molecule is slightly inclined with respect to the 001 plane of MNT. The terminal carboxylate group of the intercalated CNS is coordinated to one Na^+^, d(CO…Na) = 2.276–2.318 Å, and has hydrogen bonds with water molecules, d(CO…HOH) = 1.866 Å. The terminal ammonium group forms hydrogen bonds with the basal O atoms of the mineral surface, d(NH…OSi) = 2.283–2559 Å, and with water molecules, d(NH…OH_2_) = 1.765 Å. The lateral amino group forms also hydrogen bonds with the mineral O atom, d(NH…OSi) = 1.908 Å. The carbonyl group of the lateral amide moiety of CNS is coordinated to one Na^+^, d(CO…Na) = 2.256 Å. Some Na^+^ cations are coordinated with the mineral surface O atoms, d(Na…OSi) = 2.348–2.403 Å, with water molecules, d(Na…OH_2_) = 2.294–2.425 Å, and with the hydroxyl O atom, d(Na…OH) = 2.337 Å. The hydroxyl is oriented to the mineral surface forming a hydrogen bond, d(OH…OSi) = 1.852 Å. Water molecules also form hydrogen bonds with the mineral surface basal O atoms, d(HOH…OSi) = 1.986–2.486 Å, and with a carboxyl group, d(HOH…O=C) = 1.897 Å. The thiol group forms weak intramolecular hydrogen bonds with a carbonyl group, d(SH…O=C) = 2.819 Å, and with water molecules, d(HS…HOH) = 2.859 Å.

The intercalation energy *E*_inter_ was calculated considering the initial and final steps of this intercalation process in dry conditions:

*E*_interdry_ = *E*_MNT-CNS_ − *E*_MNT_ − *E*_CNS_ (3)

with −133.12 kcal/mol per 3 × 2 × 1 supercell. This value indicates that this intercalation is energetically favorable.

The intercalation of CNS in wet conditions can be presented as the following reaction:

MNT + CNS_80w ↔ MNT-CNS + 80w(4)

Then, considering the initial and final steps of the intercalation process, we calculated the intercalation energy of the tripeptide to the MNT system in wet conditions:

*E*_interwet_ = *E*_MNT-CNS_ + *E*_80w_ − *E*_MNT_ − *E*_CNS80w_ (5)

This energy is −35.71 kcal/mol, indicating that the intercalation of CNS in wet conditions into the confined interlayer space of MNT is still energetically favorable. Once CNS is in the interlayer space of MNT, CNS molecules are disposable for release into a physiological media. The CNS intercalated in MNT is confined in an amorphous phase, and hence, its bioavailability is higher than that of the tripeptide as a solid crystal phase. Therefore, the MNT-CNS complex is an easier disposable system and could contribute to improving the pharmacokinetic properties of the tripeptide. However, we found that the intercalation energy was much lower in wet conditions than in a non-aqueous environment. In addition, the intercalation of water molecules also will compete in an aqueous suspension depending on several experimental conditions, including the concentration, pH, and temperature. Therefore, this result is a good indication for future experiments and suggests the use of a non-aqueous solvent with lower polarity than water for the intercalation process rather than aqueous media, according to previous experimental cases [14,19].

Once the MNT-CNS is formed, the CNS molecule can be released to the physiological media in a controlled way depending on the experimental conditions of pH, ionic force, and temperature. Considering the anti-inflammatory properties of the tripeptide and its disposability to the organism, the MNT-CNS complex may be one of the most suitable pharmaceutical preparations for the system.

### 2.4. Spectroscopic Properties

**Figure 5:**
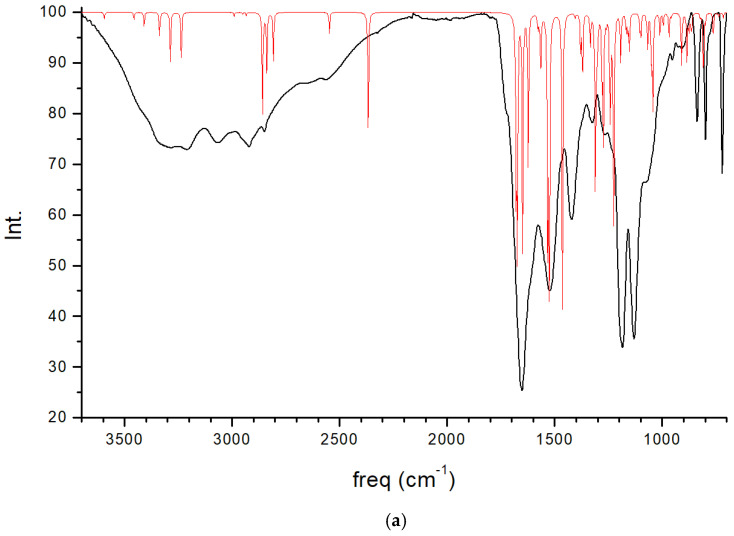

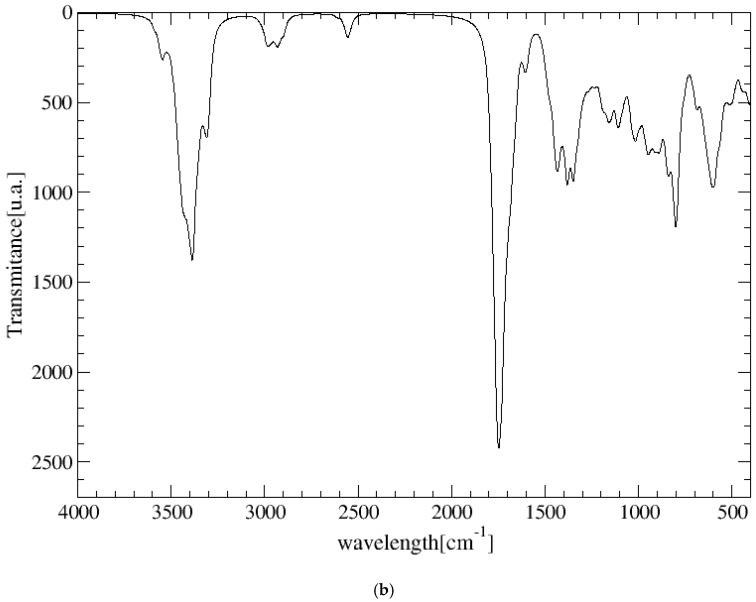
Experimental FT-IR spectrum of CNS in the solid state (in black) compared with the calculated spectrum of CNS using CASTEP (in red) (**a**), and the IR spectrum of a model of CNS in an amorphous state (**b**), obtained after molecular dynamics simulations and optimization using INTERFACE.

**Figure 6:**
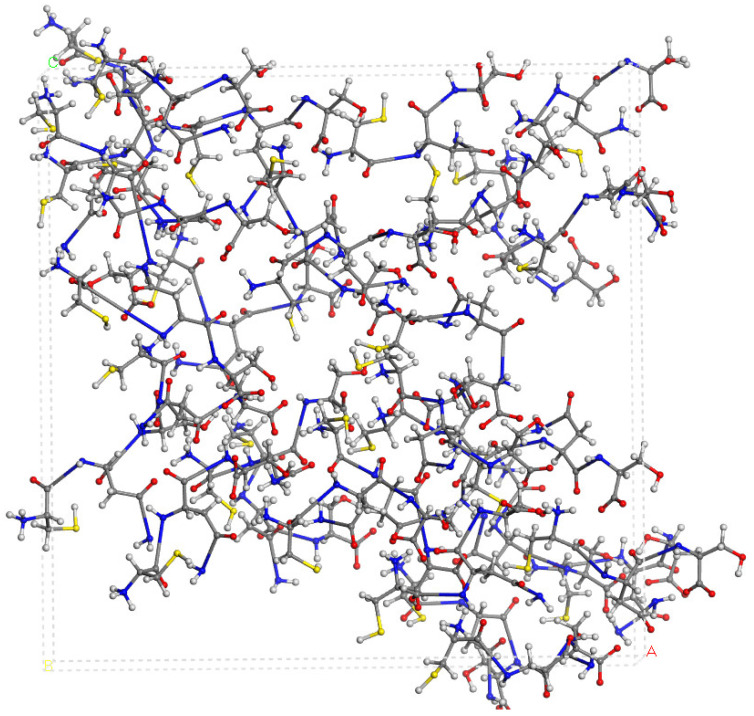
A model of the CNS in an amorphous state optimized after the molecular dynamics simulations using INTERFACE.

The experimental FT-IR spectrum of CNS is described, for the first time, in Figure 5. This is useful for comparing it with the calculated frequencies of CNS (Table 1), despite the calculated spectrum being from an isolated molecule in a vacuum at 0 K, whereas the experimental spectrum is in a solid state at room temperature with a lower resolution. Nevertheless, we can observe that the most intense bands correspond to the ν(C=O) and ν(CN) stretching and δ(NH) bending modes, which are overlapping. A broad multiple band is observed at 3500–2500 cm^−1^, assigned to the following modes: ν(NH) of the NH, NH_2_, and NH_3_^+^ groups, ν(CH) of the CH, CH_2_ groups, ν(SH) of the Cys unit, and ν(OH) of the Ser unit. The overlapping of these bands does not allow for distinguishing the bands to assign them with precision. However, our calculated frequencies of the main vibration modes allow us to assign them (Table 1, Figure 5). The high-frequency shoulder of the broad band can be assigned to the ν(NH) mode of the NH_2_ amide group of the lateral chain of Asn in both the asymmetric and symmetric modes, with the last band appearing at a lower frequency. The ν(NH) mode of the NH_3_^+^ group also appears in this frequency zone. This fact has been found also in previous works on peptides with Asn [32,33]. The ν(NH) mode of the bridging amide NH groups appears at a lower frequency. However, these frequencies can change slightly with changes in the hydrogen bond interactions produced by conformational variations that can occur at room temperature [32,33]. The ν(NH_2_) of Asn appears at higher frequencies than the Ser one. The ν(NH_3_^+^) band of the Cys unit appears at a very low frequency (2363–2367 cm^−1^) due to the strong hydrogen bond with the carboxylate group. In the experimental spectrum, CNS is in a solid state. This ammonium group would interact with other molecules, and the intensity of this band would be consequently lower. The experimental CNS solid used for the FT-IR spectrum was not recrystallized, and its crystallinity is limited. We simulated a model of the CNS solid in an amorphous state with a box of 32 CNS molecules disordered by molecular dynamics simulation with INTERFACE. The optimized structure has the ammonium groups oriented toward the carboxylate and carbonyl groups of vicinal molecules (Figure 6). Its FT-IR spectrum was simulated (Figure 5b), confirming our hypothesis that the frequency and intensity of the ν(NH_3_^+^) band decreases due to the interatomic interactions. These changes, plus the overlap with other modes, such as ν(CH), ν(OH), and ν(SH), yield a broad multiple band, as observed in the experimental spectrum at room temperature (Figure 5). The intense band at 1720–1600 cm^−1^ is assigned to the ν(C=O) mode. The lower-frequency shoulder of this band, and the band at 1570–1500 cm^−1^ can be assigned to the δ(NH_2_) and δ(OH) modes, which appear at very close frequencies. The asymmetry to the lower frequency of this band (around 1450 cm^−1^) shows an overlap with other bands that can be assigned to the peptide δ(NH) mode. The band at 1423 cm^−1^ can be assigned to the δ(CH_2_) mode. Despite the low resolution of the experimental spectrum of CNS, our calculated frequencies can be considered consistent with the experimental values of our work and similar systems of other authors (Table 1).

In the CNS intercalated into the interlayer space of the clay mineral MNT (MNT-CNS), some interesting vibrational frequency shifts were observed. The stretching ν(NH_3_^+^) bands appear at a lower frequency in the MNT-CNS (Table 2) than in the isolated molecule due to the hydrogen bond interactions with water molecules and the basal O atoms of the MNT interlayer surface. On the contrary, the ν(NH_3_^+^) band of the Cys unit, which appear at 2363 cm^−1^ in CNS molecules, appear at a higher frequency (2652 cm^−1^) in MNT-CNS because of the conformation change in the confined space of MNT, where the hydrogen bond effect decreases. A drastic frequency shift was observed in ν(OH), from 2895 cm^−1^ in the CNS molecule to 3493 cm^−1^ in MNT-CNS. The intramolecular hydrogen bond of serine disappears, forming only electrostatic interactions. During the stretching vibration mode of the OH group, the H atom moves toward the tetrahedral cavity of MNT, and the distances with the basal tetrahedral O atoms are not altered significantly. The ν(SH) of MNT-CNS appears at a higher frequency than in the isolated CNS due to the conformational change produced during the intercalation, decreasing the intermolecular interaction of the SH group. The bands of ν(C=O) appear at a lower frequency in MNT-CNS than in the isolated CNS due to the interactions in the interlayer space of MNT, coordination with the Na cations, and hydrogen bonds with water molecules. A drastic frequency difference was observed in the δ(OH) band, which appears at a lower frequency in the intercalated system than in the isolated molecule and in the experimental CNS solid. This fact is due to the lack of an intramolecular hydrogen bond of this OH group. These frequency differences can be a useful signal for experimental monitoring intercalation of this peptide into MNT. On the other hand, the intercalation of CNS in the confined MNT surface produces an increase in the δ(NH_3_) frequency of the ammonium group with respect to the isolated CNS molecule and the experimental CNS solid. The bands at frequencies lower than 1200 cm^−1^ in MNT-CNS are not useful because of the overlap with the mineral ν(Si-O) bands.

The acceptable agreement between our experimental and calculated frequencies in CNS allowed us to predict the IR spectrum of SN, whose spectrum has not been reported previously. Three situations of SN were calculated: (i) the dimer as an isolated molecule in the tautomer form; (ii) the dimer monohydrate as a molecular complex in the zwitterion form; and (iii) the crystal structure of SN. The frequencies of the main normal vibration modes of these three models were calculated (Table 2). These values were compared to the experimental data for Ser and Asn amino acids, since previous experimental IR data were not found for the SN dipeptide. Nevertheless, the calculated frequencies are consistent with the experimental data of similar compounds (Figure 5, Table 1).

In the isolated SN dipeptide, *ν*(NH_2_) appears at the highest frequencies due to the lack of intermolecular interactions (Table 2). In the monohydrate complex, the *ν*(NH_2_) bands appear at lower frequencies than in the isolated SN due to the intermolecular hydrogen bonds between the amino groups and water molecules. In the SN crystal, the *ν*(NH_2_) frequencies drastically decrease, which is due to the different intermolecular interactions where the amine groups are involved, such as the amino group with the carbonyl O atom. In all cases, the *ν*(NH_2_) band of the asparagine moiety appears at higher frequencies than the serine ligand. The *ν*(OH) band of the hydroxyl group of serine appears at the highest frequency in the isolated SN due to the lack of intermolecular interactions. This frequency decreases in the monohydrate complex, analogously to NH_2_, whereas this band appears at a much lower frequency (2867 cm^−1^) in the crystal structure due to the intermolecular interactions. However, two *ν*(OH) bands with low frequencies are observed in the isolated SN. These bands are assigned to the tautomeric form (Figure 2c): (i) the H atom between the carboxylate and the vicinal NH groups at 3203 cm^−1^; and (ii) the H atom between the carbonyl and ammonium groups at 3098 cm^−1^. In the water molecule, the *ν*(OH) band of the free O-H bond also appears at the highest frequency, 3780 cm^−1^, whereas the O-H bond forming an intermolecular hydrogen bond shows this band at a lower frequency than the previous frequency, 3629 cm^−1^. These interactions are stronger in the crystal structure, and the *ν*(OH) of this water appears at lower frequencies than in the monohydrate complex. In the *ν*(CH) bands of CH_2_, the asparagine moiety appears at a higher frequency than the serine moiety. The high frequency of the *ν*(CN) band of the isolated SN is due to the enamine N=C bond of the tautomeric form. The δ(OH) and δ(CH) bands appear at higher frequencies in the crystal structure than in the isolated SN due to the stronger intermolecular interactions in the crystal lattice.

## 3. Materials and Methods

### 3.1. Experimental Methods

The tripeptide CNS (Cys-Asn-Ser) was prepared by sequential synthesis by Pepmic (Suzhou, China), and the peptide C_10_N_4_O_6_SH_18_ was obtained, as confirmed by mass spectrometry (MH^+^ = 323 g/mol). An Agilent-6-125B mass spectrometer was used (Agilent Tech., Inc., Santa Clara, CA, USA). The purity was confirmed by HPLC (high-performance liquid chromatography) using a Boston-green 0DS-AQ column of 250 × 4.6 mm (Shanghai Boston Analytics, Inc., Shanghai, China) with an UV-VIS detector at a wavelength of 220 nm. The product was characterized directly in the solid state by FT-IR spectroscopy using a Nicolet^TM^ iS^TM^50 (Thermo Scientific^TM^, Waltham, MA, USA) spectrophotometer at room temperature. The IR spectra were analyzed with OMNIC Spectra software (Thermo Scientific^TM^, Waltham, MA, USA).

### 3.2. Models and Calculation Methodology

An aqueous solution model of the CNS tripeptide was taken from previous studies [8] as a non-ionic molecule. Then, a zwitterion form was generated (Figure 1a) using the Materials Studio package [37]. Taking into account the lack of experimental structural information of this tripeptide, the geometry of the dipeptide Ser-Asn (SN) was taken from a previous experimental X-ray diffraction report [31] of the crystal structure of L-serine–L-asparagine monohydrate. A zwitterion form of the isolated molecule of SN was extracted from this crystal structure (Figure 1b). This dipeptide, whose experimental data are known, was calculated at the same level as CNS in order to validate the CNS results. Both peptide molecules in zwitterion form were studied as isolated molecules and also embedded into separated periodical boxes of 15 × 15 × 15 Å, applying periodical boundary conditions.

Hydrated periodical boxes were built, filling the peptide boxes with water molecules until reaching a density of 1 g/cm^3^. The placement of water molecules into the box was performed with the Monte Carlo method. In order to acquire representative results because of the large number of water molecules and configurations in our periodical boxes, the Monte Carlo and the simulated annealing methods were used. These calculations based on empirical force fields randomly explore different conformations, orientations, and configurations of the molecules in our system.

A model of MNT currently used in pharmaceutical formulations, purified Veegum HS^®^ (VHS) from Vanderbilt (R.T. Vanderbilt, Inc., Norwalk, CT, USA), was generated considering the chemical composition of this material. Then, the unit cell of MNT used in our work was Na(Al_3.17_Mg_0.83_)(Si_7.83_Al_0.17_)O_20_(OH)_4_ [14]. Considering the molecular size of CNS, a model for its adsorption in MNT was designed as a supercell of 3 × 2 × 1 in a periodical structure of Na_6_(Al_19_Mg_5_)(Si_47_Al_1_)O_120_(OH)_24_. This clay mineral has small cation substitutions of Si^4+^ by Al^3+^ in the tetrahedral sheet and of Al^3+^ by Mg^2+^ in the octahedral sheet. The Mg^2+^ cations were placed in a maximal dispersion configuration, which is be the most stable configuration [38,39]. These substitutions produce an excess negative charge that is compensated for by the presence of Na^+^ cations in the interlayer space [40].

We used different theories for calculating our systems: density functional theory (DFT) and force field methods. Different approaches based on DFT have been used: (i) for the isolated peptides, the Gauss code, based on linear combination of atomic orbitals (LCAO) basis sets, was used; (ii) CASTEP was used with plane wave basis sets for the periodical systems; and (iii) Dmol^3^ based on LCAO basis sets was also used for calculating the vibrational frequencies of the periodical systems. The Compass force field was used in our calculations to generate the optimal configurations of the water molecules in the boxes with and without peptides.

For the isolated peptide molecules, the Gauss code [41] was used with the hybrid M06-2X correlation exchange functional [42,43] and the 6-311+G(d,p) basis set [44], including the SMD continuum model [45] for simulating an aqueous medium.

Three-dimensional crystal models were calculated using the CASTEP code with 3D periodical boundary conditions [46]. The method of Grimme was also used for dispersion corrections [47]. We used the generalized gradient approximation (GGA) with the Perdew–Burke–Ernzerhof exchange–correlation functional (PBE) [48]. Ultrasoft pseudopotentials and a mesh cut-off energy value of 500 Ry were used in the Γ point of the Brillouin zone of the crystal lattice. The convergence gradient in the SCF (self-consistent field) calculations was 1 × 10^−7^ in the density matrix. The convergence tolerance parameters for the optimizations were 5 × 10^−6^ eV/atom for energy, a maximum displacement of 0.0001 Å, a maximum stress of 0.02 GPa, and a maximum force of 0.01 eV/Å [37]. The frequencies of the main normal vibrational modes of the crystals were obtained from phonon calculations, which were based on the theory of density functional perturbation (DFPT) [49]. Powder X-ray diffractograms were simulated for the crystal structures with a Cu wavelength using the Reflex code [37].

Additional DFT calculations were performed using the DMol^3^ code with GGA-PBE [48] functional, employing double-zeta extended base functions, including polarization functions (DNPs) and pseudopotentials with semi-core corrections (DSPPs) [50], and Grimme dispersion corrections [47]. Frequencies of the main vibrational normal modes were calculated by force analysis from the Hessian matrix obtained by atomic finite displacements.

For large models, the INTERFACE force field (FF) was used [51], based on empirical interatomic potentials and validated in previous works [15,19]. The geometry optimization calculations were performed using the Forcite code with periodical boundary conditions within the Materials Studio package [37], applying the Ewald summation method for the electrostatic and van der Waals interactions in each periodical box The van der Waals (vdW) interactions were calculated with the Lennard–Jones potential, V(r) = ϵ[(σ/r)^12^ − 2(σ/r)^6^], with a cut-off at 15 Å. The amorphous state model of CNS was generated with a periodical box of 32 CNS molecules in a cubic crystal lattice of 34 × 34 × 34 Å^3^ (39.3 nm^3^), with the optimization and molecular dynamics simulations carried out with the NVT ensemble at 398 K, with 1 fs steps over 10 ps.

## 4. Conclusions

The molecular structure of the anti-inflammatory tripeptide CNS was optimized at the DFT level. The crystal structure and spectroscopic properties of SN were also obtained, due to the lack of experimental pairing of CNS. The DFT crystal cell parameters are very close to the experimental results of the SN monohydrated system. From this fact, the MNT crystal structure was calculated, and the intercalation of CNS into the confined nano-space of the interlayer of MNT was modeled.

From these calculations, the following results were obtained: (i) exothermic packing energy was obtained for the SN monohydrated system; (ii) the hydration energy of CNS is also exothermic; (iii) the intercalation energy of CNS into the confined interlayer space of MNT is likewise exothermic. Furthermore, the intercalation energy from the CNS system in aqueous media in MNT is also exothermic. The conformational configurations of the tripeptide CSN with interesting anti-inflammatory activity changed during the intercalation into montmorillonite.

The vibrational spectra of SN and CNS yielded frequencies in agreement with other similar systems. In CNS intercalated in the interlayer space of MNT, some frequencies change as a consequence of the intermolecular interactions with the ions and the water molecules in the interlayer space of MNT. These frequency shifts can be a useful signal for experimentally following the intercalation process of CNS in the confined interlayer space of MNT.

Furthermore, the MNT-CNS composite preparation is energetically favorable, especially in non-aqueous media. The CNS drug will be released in controlled way under any physiological media depending on the experimental conditions of the pH and concentration. Therefore, this MNT-CNS nanomaterial can be considered a good pharmacological preparation to improve the pharmacokinetic properties of CNS as a drug, ahead of CNS crystal. This indicates that this intercalation can be a promising procedure for preparing hybrid nanomaterials for therapeutic applications with controlled release properties and stability. Finally, it is also important to note that inflammatory processes are inherent to the most frequent human and veterinary diseases. This study can be extended to other oligopeptides and clay minerals.

## Figures and Tables

**Table 1:** Calculated IR frequencies (cm^−1^) of CNS as a molecule (CNSmol) and intercalated into MNT (MNT-CNS), calculated using DMol^3^ and CASTEP (in brackets) and compared with experimental values.

Mode	CNSexp *^g^*	CNSmol	MNT-CNS
ν(NH_2_)	3520–3440, 3560–3505 *^a,m^*, 3393–3383*_s_* *^a,m^*, 3430*_s_* *^n^*	3534 *^a^* (3594 *^a^*, 3456*_s_* *^a^*)	3595 *^a^*, 3449*_s_* *^a^*
ν(NH_3_^+^)	3420–3350, 3430 *^n^*, 2620–2580	3459 *^c^*, 3384 *^c^*, (3410*_as_*, 3337*_s_*) *^c^*, 2363 *^c,f^* (2367 *^c,f^*)	3401 *^c^*, 3154 *^c,d^*, 2652 *^c,e^*
*ν*(NH)	3280–3200, 3293–3238 *^o^*	3469 *^b^*, 3212 *^a,c^*, 3108 *^a,f^* (3287 *^b^*, 3236 *^a^*)	3494 *^b^*, 3385,
*ν*(CH)	3100–3063	3087 *^c^*, 3071 *^b^*, 3070 *^c^*, 3061 *^b^*, 3016 *^a^*, 2822 *^b^* (3022–3008 *^c^*, 2991 *^b^*)	3057 *^c^*, 3052 *^b^*, 3013 *^a^*
*ν*(CH_2_)	3063–2950	3078 *^a^*, 3004*_s_* *^a,c^* (2990–2975 *^a^*, 2951*_s_* *^c^*, 2934 *^b^*, 2839 *^a^*, 2808 *^b^*)	3106 *^c^*, 3078 *^b^*, 3065 *^a^*, 3042*_s_* *^c^*, 3016*_s_* *^b^*, 3004*_s_* *^a^*
*ν*(OH)_ser_	2840	2895 *^b^* (2858)	3493
*ν*(SH)	2620–2580, 2551 *^j^*, 2563 *^k^*	2622 (2548)	2656
*ν*(C=O)	1720–1600, 1704–1678 *^h^*, 1709–1683 *^m^*, 1657–1648 *^o^*	1732 *^a^*, 1713–1705 *^a,b^* (1674–1650 *^a^*)	1701 *^c^*, 1671 *^a,b^*, 1654 *^a^*, 1608 *^b,f^*
δ(OH)_ser_	1570–1500 *^b^*	1659 *^b^*, 1550 *^b^* (1624, 1525) *^b^*	1446–1406 *^b^*
δ(NH_3_)	1570–1500, 1616–1585 *^m^*	1604–1600 *^c^* (1578, 1571)	1678 *^c^*, 1621 *^c^*, 1537*_s_* *^c^*
δ(NH_2_)	1550–1500, 1612 *^h^*	1599 *^a^* (1565*_s_* *^a^*)	1572*_s_* *^a^*,
δ(NH)	1470*_sh_*, 1514 *^m^*, 1533 *^o^*	1519 *^a,b^*, 1479 *^a,c^*, 1177 (1680 *^c^*, 1533 *^b^*, 1464 *^a^*)	1510 *^a,b^*, 1458 *^a,c^,* 1179
δ(CH_2_)	1423, 1467–1450 *^i^*, 1424 *^j^*	1473*_s_* *^b^*, 1440*_s_* *^a^*, 1433*_s_* *^c^* (1448*_s_* *^b^*, 1405*_s_* *^c^*, 1380*_s_* *^a^*, 1335 *^b^*)	1484*_s_* *^b^*, 1422*_s_* *^c^*, 1404–1393*_s_* *^a^*
δ(CH)	1324, 1341 *^j^*	1353, 1340 *^a^*, 1338 *^c^*, 1299–1256 (1311 *^c^*, 1304 *^a^*, 1274 *^b^*)	1344–1224
γ(CH)	1184	1256–1254	1201–1191, 1120 *^c^*, 994 *^b^*
*ν*(C-O)	1131–1050, 1030 *^i^*	1058 *^b^* (1044 *^b^*)	
γ(NH)	800, 740–721 *^o^*	(809–799 *^b^*)	1082 *^a^*, 1108 *^c^*
δ(SH)	838	998 (995)	914 *^c^*
γ(OH)	799–722	947 (886)	

*^a^* Lateral chain of asparagine. *^b^* Serine. *^c^* Cysteine. *^d^* Hydrogen bonds with basal O atoms of mineral surface. *^e^* Hydrogen bonds with water molecule. *^f^* Forming hydrogen bonds with C=O group. *^g^* Our work in the solid state. *^h^* From [34] for Asn in an aqueous solution. *^i^* From [34] for Ser in an aqueous solution. *^j^* From [34] for Cys in an aqueous solution. *^k^* From [35] for Cys in KBr. *^m^* In Asn peptides [32]. *^n^* In Asn peptides [33]. *^o^* In other peptides [36]. *_s_* means the symmetric vibration mode; *_sh_* means the shoulder in the band.

**Table 2:** Calculated IR frequencies (cm^−1^) of SN peptide calculated with CASTEP.

Mode	Exp	SN	SN Monohydrate	SN Crystal
*ν*(NH_2_)		3566 *^a^*, 3467 *^b^*, 3438*_s_* *^a^*, 3384*_s_^b^*	3520 *^a^*, 3353 *^b^*, 3059 *^b,g^*, 3015 *^b,h^*, 2920 *^a,g^*, 2683 *^c^*	3390 *^a^*, 3284 *^a,g^*, 3249*_s_* *^a^*, 2946–2934 *^b^*, 2844–2822 *^b^*
*ν*(OH)		3740 *^b^*, 3203 *^a,f^*, 3098 *^b,d^*	3780 *^k^*, 3736 *^b^*, 3629 *^k^*	3533 *^k^*, 3406 *^k^*, 2867 *^b^*
*ν*(CH_2_)		3030 *^a^*, 2969 *^b^*, 2963*_s_* *^a^*, 2955*_s_^b^*	3004 *^a^*, 2968 *^b^*	3026 *^a^*, 2958*_s_* *^a^*
*ν*(CH)		2924 *^a^*, 2893 *^b^*	3022 *^b^*, 2962–2932 *^a^*,	3017 *^b^*, 3004 *^a,b^*
*ν*(C=O)	1704–1677 *^i^*	1756 *^e^*, 1700 *^a^*	1680, 1670 *^a^*	
*ν*(C-N)		1690		1090 *^a^*, 1058 *^b^*
δ(NH)	1622–1612 *^i^*	1599*_s_* *^b^*, 1570*_s_* *^a^*	1663 *^a^*, 1625 *^b^*, 1585 *^a^*, 1504 *^b^*	1649–1639 *^b^*, 1630 *^a^*,1609 *^b^*, 1577*_s_* *^a^*, 1551–1534, 1506_s_ *^b^*
δ(CH_2_)	1467–1450 *^j^*	1450*_s_* *^b^*, 1419*_s_* *^a^*	1455 *^b^*, 1402 *^a^*	
δ(OH)	1420–1181 *^j^*	1404 *^g^*, 1401 *^b^*	1596 *^k^*, 1190 *^b^*	1617 *^k^*, 1469 *^b^*
δ(CH)		1334 *^a^*, 1309 *^b^*	1334 *^b^*, 1302–1256 *^a^*	1432*_s_* *^b^*, 1419*_s_* *^a^*, 1362*_s_* *^b^*, 1390–1272
γ(CH)	1382–1170 *^j^*			1261, 1171
γ(NH)				1226, 1113
*ν*(C-O)	1030 *^j^*			1051 *^b^*
γ(OH)	940 *^j^*			982 *^b^*

*^a^* Lateral chain of asparagine. *^b^* Serine. *^c^* NH of the central amide group. *^d^* Enolic form vicinal to the ammonium group of serine. *^e^* Carboxylate group. *^f^* Carboxylate forming a strong hydrogen bond with a NH group. *^g^* N-H bond oriented to the carbonyl group. *^h^* N-H bond forming a hydrogen bond with water molecules. *^i^* From [34] for Asn in an aqueous solution. *^j^* From [34] for Ser in an aqueous solution. *^k^* Water. *s* means the symmetric vibration mode.

## Data Availability

The datasets generated for this study are available upon request to the corresponding author.

## Supplementary Materials

The following supporting information can be downloaded at: https://www.mdpi.com/article/10.3390/molecules29174250/s1, Table S1: Main geometrical parameters (distances in Å, and angles in º) of CNS and SN peptides in zwitterion forms (numbering is based on Figure 1) optimized as isolated molecules with Gaussian and Castep (in brackets); Table S2: Atomic coordinates of the optimized CNS molecule in zwitterion form; Table S3: Atomic coordinates of the optimized molecular structure of SN in zwitterion form; Table S4: Atomic coordinates of the optimized molecular structure of SN in tautomeric form; Figure S1: Powder X-ray diffractograms of the SN peptide simulated for the experimental crystal structure (black) and the optimized one (red).
