# Supplementary material for: Structure and Intercalation of Cysteine–Asparagine–Serine Peptide into Montmorillonite as an Anti-Inflammatory Agent Preparation—A DFT Study
Source: Molecules. 2024 Sep 7;29(17):4250. doi: 10.3390/molecules29174250 (PMC11396832; doi:10.3390/molecules29174250)
Supplement: Supplementary file 1 [file molecules-29-04250-s001.zip › molecules-3141469-supplementary.pdf]

SUPPLEMENTARY MATERIAL

Structure and Intercalation of Cysteine–Asparagine–Serine Peptide into Montmorillonite as an Anti-Inflammatory Agent Preparation—A DFT Study

Carolina Barrientos-Salcedo [1], Catalina Soriano-Correa [2,3,*], Alfonso Hernández-Laguna [2] and Claro Ignacio Sainz-Díaz [2,*]

[1] Laboratorio de Química Médica y Quimiogenómica, Universidad Veracruzana, Veracruz C.P. 91700, Mexico; cabarrientos@uv.mx
[2] Instituto Andaluz de Ciencias de la Tierra, Consejo Superior de Investigaciones Científicas, Av. de las Palmeras, 4, 18100 Armilla, Granada, Spain; a.h.laguna@csic.es
[3] Unidad de Química Computacional, Facultad de Estudios Superiores Zaragoza, Universidad Nacional Autónoma de México, Iztapalapa, Mexico City C.P. 09230, Mexico
* Correspondence: csorico@comunidad.unam.mx (C.S.-C.); ci.sainz@csic.es (C.I.S.-D.)

Table S1. Main geometrical parameters (distances in Å, and angles in º) of CNS and SN peptides in zwitterion forms (numbering is based on Figure 1) optimized as isolated molecules with Gaussian and Castep (in brackets).

Geometric Parameter	Cys-Asn-Ser (CNS)	Ser-Asn (SN)
N1-H2	1.022 (1.027)	
N15-H19		1.026 (1.808)
S37-H39	1.349 (1.357)	
C4=O9 (ASN)	1.224 (1.239)	
C7=O13 (SER)		1.239 (1.341)
C4-N1	1.354 (1.349)	
C7-N15		1.350 (1.282)
C27-N31 (NH_3^+)	1.467 (1.517)	
C3-N1 (NH_3^+)		1.503 (1.476)
C8-C17 (lateral chain, ASN)	1.559 (1.546)	1.539 (1.545)
C22=O25 (lateral chain, ASN)	1.229 (1.230)	1.244 (1.220)
C22-N26 (NH_2) (lateral chain, ASN)	1.355 (1.368)	1.349 (1.379)
C7-O14 (O-C-O)	1.343 (1.246-1.269)	
C8-O9 (O-C-O)		1.268 (1.209, 1.345)
C3-C6 (-COH)	1.534 (1.546)	1.548 (1.531)
H2···O25	2.010 (1.963)	
H19···O25		(0.993)
H2N1C4C8	169.8 (1.0)	
H19N15C7C3		169.2 (127.6)
O28C23N15C8	5.0 (5.3)	
N1C4C8N15	177.1 (179.4)	
O25C22C17C8	81.0 (22.7)	55.6 (56.1)
N26C22C17C8	96.1 (157.6)	128.7 (128.3)
C4N1C3C7	69.0 (73.2)	

C23N15C8C4	169.5 (161.4)	
C6C3N1C4	170.2 (163.6)	
C6C3C7N15		86.2 (117.4)

Table S2. Atomic coordinates of the optimized CNS molecule in zwitterion form.

Atom	x	y	z
N	-0.33917000	0.32305300	-0.15170800
H	-0.48611300	0.81711400	0.72441100
C	1.02788700	0.10031800	-0.59133800
C	-1.30811300	-0.51903400	-0.53085900
H	1.10426300	0.26582500	-1.66738400
C	1.90773400	1.10304800	0.14616100
C	1.49522800	-1.34369400	-0.27745200
C	-2.57789800	-0.48113900	0.31022000
O	-1.19027900	-1.31415000	-1.46288200
H	1.56442100	2.11731300	-0.07995100
H	1.81635500	0.93182300	1.22571100
O	3.24841900	0.91802100	-0.27990100
O	1.07962900	-1.85960900	0.78725500
O	2.28002600	-1.86689900	-1.10297200
N	-3.66977900	-0.96912800	-0.51087000
H	-2.79707200	0.54244900	0.61805000
C	-2.39726200	-1.36711900	1.55322700
H	3.80099300	1.55051500	0.19115700
H	-3.43549500	-1.52056000	-1.32974600
H	-3.38058800	-1.52819600	2.00635200
H	-1.99973600	-2.34469100	1.26777400
C	-1.52699400	-0.73718500	2.62198900
C	-4.94005600	-0.70467200	-0.21105500
H	-7.77744200	-2.28479600	-0.95818700
O	-1.30922700	0.47819700	2.65986500
N	-1.05359400	-1.58104300	3.54456600
C	-5.98854300	-1.22777200	-1.18921200
O	-5.30126100	-0.06401000	0.77492800
H	-1.21255900	-2.57543100	3.47285800
H	-0.49221400	-1.22462900	4.30586600
N	-6.98638300	-1.98804000	-0.37722500
H	-5.56250400	-1.91810600	-1.91541100
C	-6.71840300	-0.07596000	-1.87266600
H	-7.34725000	-1.39774700	0.38225800
H	-7.46179300	-0.48308000	-2.55924300
H	-7.22745200	0.53838900	-1.12869600
S	-5.61571900	0.96358800	-2.87653500
H	-6.56692300	-2.82490100	0.04217000
H	-5.00313400	1.56994000	-1.84152700

Table S3. Atomic coordinates of the optimized molecular structure of SN in zwitterion form.

Atom	x	y	z
O	3.65792100	6.94836500	2.92696800
H	4.37698200	7.43145000	2.50448900
O	3.79017400	2.60028300	3.83021000
O	3.10666500	-1.23351800	2.31867700
O	0.97662200	2.47820300	-0.03924800
O	1.42806900	0.28518600	-0.09913100
N	3.42680800	5.01905300	4.89294600
H	2.79088100	4.65436100	5.60777100
H	3.56221700	6.02288100	5.05321100
H	4.32808500	4.53894300	4.99905000
N	2.15570200	2.86327000	2.27832400
H	1.55379900	3.51168600	1.78061500
N	0.93529000	-1.80459300	2.53760400
H	1.14098300	-2.78329100	2.39016100
H	-0.02383700	-1.51604400	2.65788000
C	2.89275600	4.79554000	3.52357400
H	1.86122900	5.14640100	3.51012500
C	3.73477900	5.58802300	2.53476200
H	4.76878600	5.22980300	2.56398500
H	3.32912300	5.43703000	1.53139200
C	2.97755000	3.30238300	3.22712100
C	2.19125400	1.49828400	1.78253400
H	3.23374400	1.20750900	1.62804000
C	1.54672100	0.52764500	2.78107900
H	1.92210500	0.75311200	3.78360300
H	0.46247400	0.65520400	2.78691100
C	1.93017900	-0.90852900	2.50614600
C	1.46893900	1.42336100	0.42320200

Table S4. Atomic coordinates of the optimized molecular structure of SN in tautomeric form.

Atom	x	y	z
O	3.64629800	6.93580800	2.90391600
H	4.24188400	7.43273200	2.33264400
O	3.80332800	2.54228000	3.70524100
O	3.08473300	-1.26406200	2.34024500
O	1.17409200	2.42405700	-0.18443000
O	1.58978200	0.25225500	-0.16971000
N	3.57451300	4.85937600	4.82799300
H	2.89878900	4.78495100	5.58202300
H	4.01868400	5.76816500	4.91485000
N	2.13477000	2.89125600	2.16781500
N	0.88740200	-1.76150500	2.46631800
H	1.06486200	-2.74717400	2.32755400
H	-0.06420100	-1.44137500	2.56557800
C	2.90244300	4.76692800	3.53247500
H	1.86744700	5.11578800	3.55961600
C	3.66692000	5.57615300	2.49123900
H	4.69803100	5.20997300	2.42982800
H	3.18628600	5.45663200	1.51757900
C	2.90595200	3.31384200	3.08713800
C	2.26682600	1.49948700	1.76899500
H	3.31630700	1.20107100	1.67501900
C	1.56788500	0.54704600	2.75133000
H	1.93076100	0.77028800	3.75791400
H	0.48909500	0.71028800	2.73165700
C	1.91375800	-0.90269500	2.48523900
C	1.65217900	1.32487700	0.38801000
H	4.20433500	3.11915500	4.39702200
H	1.30501100	3.16023900	0.44939500

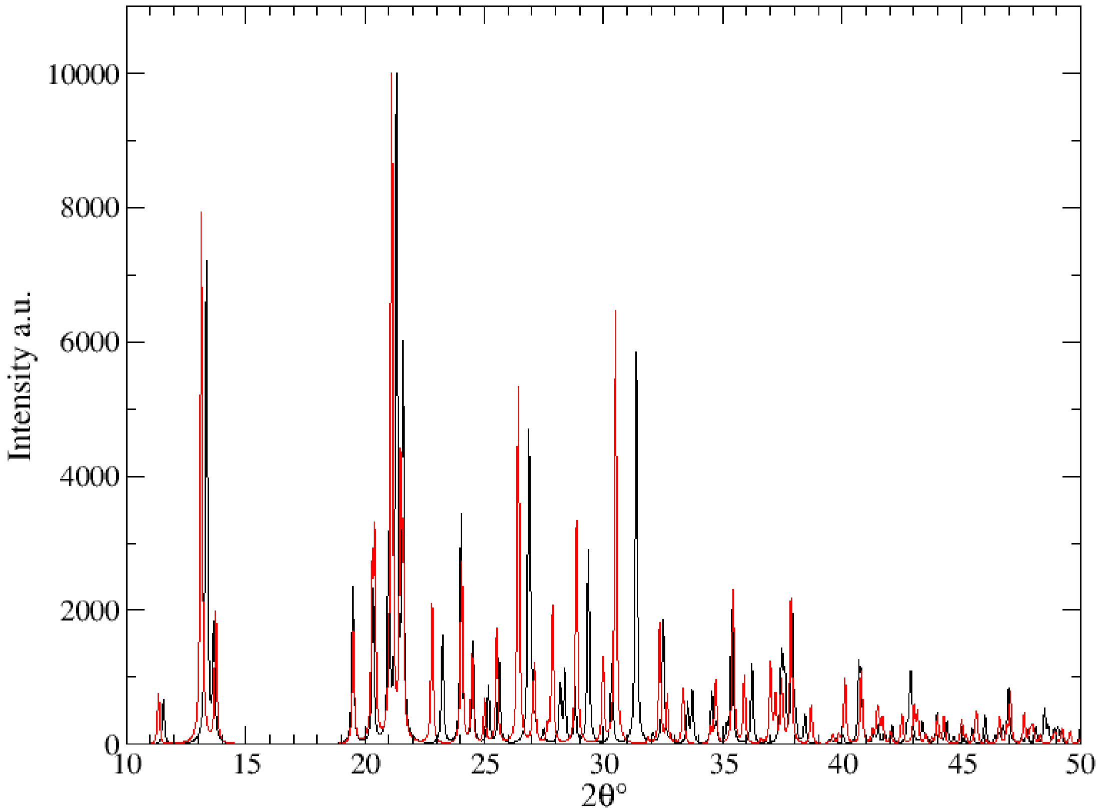

Figure S1. Powder X-ray diffractograms of the SN peptide simulated for the experimental crystal structure (black) and the optimized one (red).
